# Extracellular Redox Balance as a Determinant of Immune Regulation and Tissue Inflammation

**DOI:** 10.3390/antiox15030280

**Published:** 2026-02-24

**Authors:** Rebecca Seitz, Martina Müller, Karsten Gülow

**Affiliations:** Department of Internal Medicine I, Gastroenterology, Hepatology, Endocrinology, Rheumatology, Immunology, and Infectious Diseases, University Hospital Regensburg, 93053 Regensburg, Germany; rebecca.seitz@ukr.de (R.S.); martina.mueller-schilling@ukr.de (M.M.)

**Keywords:** reactive oxygen species (ROS), extracellular redox regulation, immune cell activation, inflammation, oxidative stress

## Abstract

Reactive oxygen species (ROS) are widely recognized as intracellular signaling mediators and contributors to oxidative damage. Increasing evidence indicates that redox processes in the extracellular space constitute a distinct and functionally relevant layer of immune regulation. Extracellular ROS are generated in a spatially and temporally controlled manner by immune and non-immune cells and are shaped by local antioxidant buffering, redox-active metabolites, and tissue architecture. Rather than acting as diffuse by-products of inflammation, extracellular redox conditions modulate immune cell activation, migration, and intercellular communication by influencing surface-associated signaling events and receptor responsiveness. Physiological redox control in the extracellular compartment supports host defense, tissue repair, and coordinated immune responses. In contrast, disruption of spatial redox regulation promotes chronic inflammation, immune dysfunction, cancer-associated immune suppression, and systemic inflammatory states such as sepsis. Loss of redox confinement and insufficient extracellular buffering uncouple redox signaling from its regulatory function and contribute to endothelial dysfunction, immune dysregulation, and tissue injury. Together, these observations identify the extracellular redox balance as an integral component of immune regulation with important implications for understanding inflammatory pathology and for the development of strategies that preserve localized redox control rather than globally suppressing ROS.

## 1. Introduction

The rise in atmospheric oxygen (O_2_) through oxygenic photosynthesis fundamentally reshaped life on Earth [1,2,3,4]. Adaptation to these conditions required the evolution of antioxidant defense systems that tightly regulate reactive oxygen species (ROS) while preserving O_2_-dependent energy production. As a result, redox regulation became embedded in the core biology of all aerobic cells [5]. Although ROS can cause cellular damage, they also function as physiological signaling molecules. Their dual role—cytotoxic at high concentrations yet regulatory at controlled levels—reflects this evolutionary integration.

The integration of redox processes into fundamental signaling pathways is especially evident in the immune system. Immune cells rely on redox-dependent regulatory mechanisms to control activation, differentiation, cytokine production, and effector functions. Mitochondria and NADPH oxidases generate ROS during antigen recognition, phagocytosis, or inflammatory responses, and these ROS act both intracellularly and, in part, extracellularly to shape immune outcomes [3,4,5,6,7,8,9,10,11]. Extracellular ROS, extracellular antioxidant enzymes, thiol availability, and redox-active metabolites can modulate receptor sensitivity, affect cytokine and chemokine function, and influence the organization of inflammatory responses in tissues. These mechanisms are increasingly recognized as critical determinants of the balance between protective immunity and pathological inflammation.

## 2. Biologically Relevant ROS

ROS comprise a diverse group of O_2_-derived molecules that differ markedly in their chemical reactivity, stability, and biological functions. Major ROS relevant for redox biology and immune regulation include superoxide anion (O_2_^•^^−^), hydrogen peroxide (H_2_O_2_), hydroxyl radicals (^•^OH), hydroperoxyl radical (HOO^•^), and lipid-derived peroxyl and alkoxyl radicals (ROO^•^ and RO^•^), as well as singlet oxygen, ozone, and hypochlorite [7,12,13,14,15,16,17,18,19].

O_2_^•−^ is typically generated by the one-electron reduction in molecular oxygen in mitochondria and by NADPH oxidases at cellular membranes. Because of its short half-life and limited membrane permeability, O_2_^•−^ acts locally and is rapidly converted to H_2_O_2_ through spontaneous- or superoxide dismutase (SOD)-catalyzed dismutation [4,12,13,14,20]. H_2_O_2_ is less reactive but more stable than O_2_^•−^, can diffuse across membranes, and serves as a key redox-signaling molecule by oxidizing protein thiols in a reversible manner [4,12,13,14,20]. At higher concentrations, however, H_2_O_2_ can react with transition metals such as Fe^2+^ to generate ^•^OH through the Fenton reaction (Fe^2+^ + H_2_O_2_ ->Fe^3+^ + ^•^OH + OH^−^). ^•^OH are highly reactive, indiscriminately modifying proteins, lipids, and nucleic acids. Due to their high reactivity, ^•^OH act as initiators of oxidative damage and play a central role in lipid peroxidation and regulated cell death pathways [13,21,22,23]. HOO^•^ is the protonated form of superoxide. Although it represents only a minor fraction at physiological pH, it becomes more relevant in acidic microenvironments or in hydrophobic compartments, where it exhibits greater membrane solubility and contributes to oxidative chain reactions. Peroxyl radicals (ROO^•^/LOO^•^) arise during lipid peroxidation when lipid radicals generated by reactive species react with oxygen [24,25,26,27]. These radicals propagate lipid peroxidation by converting polyunsaturated fatty acids into lipid hydroperoxides, which decompose into reactive aldehydes that form adducts with proteins and nucleic acids [24,25,26,28,29,30,31,32]. Additional ROS, including singlet oxygen [19,33], ozone [34], and hypochlorite [35,36], contribute to oxidative stress and immune regulation.

Together, these ROS form a dynamic network of oxidants that participate in redox signaling, host defense, and tissue injury. Their distinct chemical properties determine whether they act as regulators of physiological signaling processes or as drivers of oxidative damage and inflammation.

## 3. Sources of ROS Production

ROS are increasingly recognized not only as intracellular signaling molecules but also as extracellular mediators of communication and damage within tissues. Once released into the extracellular space, ROS can influence neighboring cells, modify the extracellular matrix, and shape tissue-level responses.

### 3.1. Mitochondria: An Indirect Source of Extracellular ROS

Mitochondria generate ROS at Complex I and III of the electron transport chain (ETC), both as by-products of electron transfer and as regulated redox signals. In immune cells, mitochondrial ROS primarily act as intracellular signaling mediators rather than direct extracellular oxidants [3,37,38,39,40]. O_2_^•−^ produced at the ETC remains confined to the mitochondrial matrix or intermembrane space and is rapidly converted to H_2_O_2_ by SOD2 [20,41,42]. Although H_2_O_2_ is membrane-permeable and can diffuse into the cytosol, it is normally buffered by peroxiredoxins and glutathione peroxidases [5,16,43]. Only when ROS production exceeds antioxidant capacity can mitochondrial H_2_O_2_ escape cellular containment and indirectly contribute to extracellular ROS levels, thereby influencing neighboring cells and tissue redox states [44,45].

### 3.2. NADPH Oxidases as the Main Source of Extracellular ROS

The NADPH oxidase (NOX) family comprises transmembrane enzymes specialized for regulated ROS production. Unlike other cellular ROS sources, NOX enzymes generate ROS as their primary function rather than as metabolic by-products [46,47]. By transferring electrons from cytosolic NADPH across membranes to molecular oxygen, they produce O_2_^•−^ or H_2_O_2_ at the extracellular or luminal side of membranes, thereby constituting the principal and spatially controlled source of extracellular ROS in immune and inflammatory contexts [46,47,48,49].

Among the NOX isoforms, NOX2 represents the quantitatively dominant source of extracellular ROS during inflammation. Highly expressed in phagocytes—including neutrophils, macrophages, monocytes, and dendritic cells—NOX2 is activated through stimulus-dependent assembly with cytosolic subunits, enabling tightly regulated but high-amplitude ROS production [50,51,52,53,54,55]. Upon activation, NOX2 generates O_2_^•−^ at the plasma or phagosomal membrane, driving the oxidative burst essential for antimicrobial defense, as illustrated by chronic granulomatous disease caused by NOX2 deficiency [52,53,56,57,58,59]. Beyond pathogen killing, NOX2-derived ROS shape the inflammatory microenvironment and account for the majority of extracellular ROS in inflamed tissues. While indispensable for host defense, excessive or sustained NOX2 activation promotes extracellular matrix oxidation, lipid peroxidation, DNA damage, and amplification of NF-κB-dependent inflammatory signaling, thereby contributing substantially to tissue injury [35,53,54,60,61,62].

Other NOX isoforms contribute to extracellular ROS generation in a tissue-specific manner. NOX1 regulates epithelial proliferation and barrier function in the gastrointestinal tract but promotes inflammation and tumorigenesis when chronically activated [50,63,64,65,66,67,68,69,70,71,72,73,74]. NOX3 is largely restricted to the inner ear and is essential for vestibular function, with minimal relevance outside this tissue [50,64,65,75,76,77,78,79,80]. NOX4 differs functionally by being constitutively active and primarily intracellular, predominantly generating H_2_O_2_ and regulating redox signaling rather than serving as a major extracellular ROS source [63,81,82,83,84,85,86,87,88]. NOX5 links calcium signaling to extracellular ROS release via EF-hand domains and modulates vascular and inflammatory signaling [89,90,91,92,93,94,95,96,97,98]. The dual oxidases DUOX1 and DUOX2 are specialized epithelial NADPH oxidases that generate H_2_O_2_ directly at the apical membrane. DUOX-derived extracellular H_2_O_2_ supports mucosal host defense, epithelial–microbial communication, and wound responses but contributes to barrier dysfunction and chronic inflammation when dysregulated [99,100,101,102,103,104,105,106].

Although multiple NOX isoforms generate extracellular ROS in a tissue-specific manner, NOX2 predominates in inflammatory contexts and quantitatively defines extracellular oxidative stress in inflamed tissues.

### 3.3. Lipoxygenases as Indirect Modulators of Extracellular ROS

Lipoxygenases (LOX) do not directly generate ROS but contribute to extracellular oxidative stress through lipid peroxidation. They catalyze the oxygenation of polyunsaturated fatty acids, generating lipid hydroperoxides (LOOHs) that decompose into reactive radical intermediates, thereby amplifying extracellular oxidative reactions [26,107,108,109,110]. LOX-derived lipid hydroperoxides functionally interact with NADPH oxidases, forming feed-forward loops that enhance extracellular ROS accumulation during inflammation [111,112]. Through this crosstalk, LOX and NOX cooperatively shape the magnitude and persistence of extracellular oxidative stress, while LOX-derived lipid mediators additionally regulate redox-dependent inflammatory signaling, immune cell recruitment, and tissue remodeling [113,114,115,116].

### 3.4. Cyclooxygenases as Indirect Regulators of the Extracellular Redox Homeostasis

Cyclooxygenases (COX-1 and COX-2) catalyze the conversion of arachidonic acid into prostaglandin endoperoxides, thereby regulating prostanoid-mediated inflammatory and homeostatic processes. Although COX enzymes do not directly generate extracellular ROS, they can indirectly modulate extracellular oxidative stress through the formation of lipid peroxides and redox-active intermediates during prostanoid biosynthesis [117,118]. COX activity is functionally linked to NADPH oxidases, as NOX-derived ROS promote COX-2 expression, while COX-derived prostanoids enhance signaling pathways that secondarily increase NOX activity, thereby shaping extracellular redox environments during inflammation [119,120].

### 3.5. Xanthine Oxidase as Source of Extracellular ROS

Xanthine oxidase (XO) is an enzyme of the purine metabolism that catalyzes the oxidation of hypoxanthine and xanthine to uric acid. Physiologically, it predominates as xanthine dehydrogenase (XDH), transferring electrons to NAD^+^. During inflammation, hypoxia, or ischemia–reperfusion, XDH is converted to XO via sulfhydryl oxidation or limited proteolysis, redirecting electron transfer to molecular oxygen and generating O_2_^•−^ and H_2_O_2_ [121,122,123]. XO can be released or bind to endothelial surfaces and extracellular matrix components, thereby acting as a direct source of extracellular ROS [124,125,126]. While tightly controlled under basal conditions, increased XO release and endothelial binding in pathological states elevate extracellular ROS and promote oxidative tissue injury [123,124,127].

## 4. The Antioxidant Defense Systems

To preserve physiological ROS signaling while preventing oxidative damage to proteins, lipids, and nucleic acids, cells rely on a tightly regulated antioxidant network that maintains redox homeostasis rather than completely eliminating ROS. This system comprises enzymatic and non-enzymatic mechanisms with high specificity for distinct ROS and spatial control across intracellular and extracellular compartments [3,5,128].

SODs constitute the primary defense against O_2_^•−^ by catalyzing its dismutation to H_2_O_2_. Mammalian cells express three isoforms with distinct localization: cytosolic SOD1, mitochondrial SOD2, and the secreted extracellular SOD3 [129,130]. SOD3 binds to heparan sulfate proteoglycans in the extracellular matrix and on endothelial surfaces, enabling efficient scavenging of extracellular O_2_^•−^, preservation of nitric oxide bioavailability, and modulation of redox signaling in vascular and inflammatory microenvironments [129,131,132,133,134].

H_2_O_2_ generated by SOD activity is further detoxified by catalase and peroxidase-based systems. Catalase, predominantly localized to peroxisomes, efficiently converts H_2_O_2_ into water and oxygen and thereby limits intracellular H_2_O_2_ accumulation and its diffusion into the extracellular space, indirectly constraining paracrine redox signaling [135,136,137,138].

Thiol-dependent redox systems provide an additional layer of redox control. The thioredoxin (Trx) system, sustained by NADPH-dependent thioredoxin reductases, regulates protein thiol redox states and supports H_2_O_2_ detoxification via peroxiredoxins [5,139]. Beyond its intracellular functions, Trx1 can be released into the extracellular space under conditions of stress or inflammation, where it modulates the redox state of cell surface proteins, cytokines, and receptors, thereby influencing inflammatory signaling in a context-dependent manner [140,141,142].

Glutathione (GSH) represents the most abundant low-molecular-weight thiol antioxidant and a central regulator of cellular redox homeostasis. While predominantly intracellular, measurable levels of glutathione are present in extracellular fluids, including plasma and epithelial lining fluids, where it contributes to ROS detoxification and redox buffering, albeit at lower concentrations and with a more oxidized redox potential compared to intracellular pools [143,144,145,146,147,148].

In the circulation, plasma proteins form a major extracellular redox buffer. Albumin provides most free thiols via its conserved Cys34 residue, which undergoes reversible oxidation and thiol–disulfide exchange [149,150,151,152]. Immunoglobulins and transferrin further stabilize extracellular redox conditions by binding redox-active metals and participating in the plasma thiol/disulfide network rather than directly scavenging ROS [153,154,155].

Low-molecular-weight antioxidants, including ascorbate, urate, and α-tocopherol, further support extracellular redox balance. Ascorbate acts as a major aqueous antioxidant, urate contributes to plasma antioxidant capacity, and α-tocopherol protects against lipid peroxidation in lipoprotein compartments [156,157,158,159,160,161,162].

## 5. Principles of Extracellular Redox Control in Immunity

Redox regulation of immune responses has predominantly been investigated within intracellular signaling networks and tissue-specific regulatory frameworks, where ROS act as tightly controlled modulators of signaling pathways and cellular fate decisions. Building on these established concepts, extracellular redox regulation introduces an additional spatial layer of control that operates at cell surfaces and tissue interfaces. Rather than replacing intracellular redox models, this perspective extends redox regulation beyond the cell, emphasizing spatial confinement, intercellular redox gradients, and extracellular buffering as determinants of immune activation and inflammatory organization.

The antioxidant defense systems described above do not abolish extracellular ROS generation/accumulation but instead confine it in space, time, and chemical identity. This spatial and temporal restriction arises from localized ROS production at defined cellular or tissue-associated sites, limited diffusion in the extracellular space, and buffering by extracellular antioxidant systems [132,163]. As a result, extracellular ROS activity is confined to discrete regions at cell surfaces, within intercellular clefts, or along tissue interfaces, where oxidant concentrations can transiently reach signaling-competent levels without spreading diffusely into surrounding tissue.

Within such confined environments, extracellular ROS act within localized re dox microdomains that enable selective modulation of redox-sensitive targets while limiting nonspecific oxidative damage [164]. The biological consequences of these microdomains depend on concentration and exposure time. Low to moderate oxidant levels favor reversible redox modifications that support signaling, whereas higher or sustained oxidant exposure exceeds local buffering capacity and drives irreversible oxidative damage [165]. These concentration- and time-dependent boundaries define functional redox thresholds that separate regulatory extracellular redox signaling from pathological oxidative stress.

While intracellular redox signaling has been extensively characterized, the functional principles by which extracellular ROS influence immune cell behavior are only beginning to be understood. Rather than acting as diffuse by-products of inflammation, extracellular ROS form spatially and temporally restricted redox environments that actively shape immune activation, communication, and tissue responses [3,8,60,166,167].

### 5.1. Spatial and Chemical Constraints of the Extracellular Redox Signaling

Extracellular redox signaling is fundamentally constrained by the chemical reactivity, diffusion properties, and short half-lives of ROS. In contrast to intracellular ROS, which act within relatively confined and buffered compartments, extracellular ROS operate in an open and heterogeneous environment characterized by variable antioxidant capacity, extracellular matrix composition, and dynamic cellular interfaces. Therefore, the biological impact of extracellular ROS is not determined by their absolute concentration but by their spatial confinement, temporal availability, and proximity to redox-sensitive targets.

Most extracellular ROS exhibit limited diffusion ranges due to rapid consumption by antioxidants, metal-catalyzed reactions, and interactions with extracellular proteins and matrix components. O_2_^•−^, in particular, is highly membrane-impermeable and short-lived, restricting its effects to the immediate vicinity of its site of generation [4,168]. H_2_O_2_, while more stable and membrane-permeable, is likewise subject to rapid enzymatic and non-enzymatic degradation in the extracellular space [4,168,169]. Extracellular ROS are therefore typically generated at defined cellular sites, such as the plasma membrane or pericellular space, and their signaling effects remain confined to local microdomains. In immune and inflammatory contexts, these sites are predominantly defined by membrane-associated NADPH oxidases, particularly NOX2 in activated phagocytes and DUOX1/2 at epithelial barrier surfaces, whereas enzymes such as xanthine oxidase contribute mainly under pathological conditions involving tissue damage, hypoxia, or ischemia–reperfusion injury [170]. These properties ensure that extracellular ROS signals remain highly localized, favoring the formation of redox microdomains at cell surfaces, within intercellular clefts, and along tissue interfaces [14,168].

The extracellular matrix plays a critical role in shaping these redox microenvironments. Matrix components such as proteoglycans, glycoproteins, and collagen fibers provide binding sites for ROS-generating enzymes, antioxidant proteins, and metal ions, thereby influencing both ROS production and decay [171,172]. The immobilization of enzymes such as NOX or extracellular SOD3 at defined matrix or membrane-associated sites promotes directional redox signaling and limits nonspecific oxidative damage [49,171,172,173,174]. In parallel, redox-active metal ions and oxidizable matrix components act as sinks or amplifiers for extracellular oxidative reactions. Together with local antioxidant buffering systems, these features determine whether extracellular ROS support regulated signaling or shift toward damaging oxidative stress. This modulates the range and persistence of redox signals [171,172,175,176].

In addition to spatial constraints, extracellular redox signaling is strongly controlled by the chemical identity of the oxidant species involved. O_2_^•−^, H_2_O_2_, and LOO^•^ differ markedly in their target specificity, reaction kinetics, and reversibility of modifications [12,177]. In contrast, H_2_O_2_ preferentially oxidizes reactive cysteine residues in a reversible manner, enabling redox-based signal transduction [15,106,178,179,180]. Highly reactive species such as O_2_^•−^ and ^•^OH induce rapid and largely irreversible oxidative modifications that favor tissue injury over signaling [34,181,182,183]. Accordingly, extracellular redox imbalance arises when high-amplitude O_2_^•−^ production by enzymes such as NOX2 or xanthine oxidase overwhelms local antioxidant buffering, whereas sustained H_2_O_2_ generation by DUOX1/2 or NOX1 more often supports chronic redox signaling at epithelial and tissue interfaces. Supporting evidence shows that excessive ROS production by NADPH oxidases is linked to oxidative stress in chronic diseases, and that xanthine oxidase is a major ROS source in ischemia–reperfusion injury and related oxidative damage [184].

Temporal control represents an additional layer of regulation. Extracellular ROS are often generated in short, stimulus-coupled bursts, for example, during immune cell activation or epithelial stress responses [3,14,35,185,186]. Such transient ROS production can convey information without overwhelming local antioxidant defenses. In contrast, sustained or repetitive extracellular ROS generation leads to progressive oxidation of extracellular targets, depletion of antioxidant buffering capacity, and a shift from adaptive redox signaling to pathological oxidative stress [3,14,187,188,189].

Together, these spatial, chemical, and temporal constraints define extracellular redox signaling as a highly localized and tightly regulated process. Rather than functioning as freely diffusing mediators, extracellular ROS operate within restricted microenvironments that enable selective modulation of immune cell activation and tissue responses while limiting collateral damage.

### 5.2. Spatial Organization of Inflammation by Extracellular Redox Gradients

Inflammatory responses are characterized by pronounced spatial heterogeneity, and extracellular redox reactions contribute directly to this organization by generating redox gradients within tissues. Rather than being uniformly distributed, extracellular ROS accumulate in defined regions of injury, infection, or cellular activation. These localized accumulations generate concentration gradients that provide immune cells with positional and contextual information. These gradients arise from the localized activity of ROS-producing enzymes, restricted diffusion, and uneven distribution of antioxidant buffering capacity within tissues (Table 1) [14,35,60,167,190].

At sites of tissue damage or microbial invasion, distinct ROS-generating enzyme systems dominate depending on the cellular and pathological context. NOX2 activation in infiltrating phagocytes represents the principal source of high-amplitude extracellular O_2_^•−^ during acute inflammation and infection, whereas DUOX1/2 activity at epithelial interfaces generates more spatially confined H_2_O_2_ gradients associated with barrier defense and repair. Under conditions of hypoxia, ischemia–reperfusion injury, or extensive tissue damage, the release and endothelial surface binding of xanthine oxidase provides an additional source of extracellular ROS. Together, these enzyme systems generate steep extracellular redox gradients that typically extend over micrometer-scale distances and decay rapidly with increasing distance from the source, reflecting both chemical instability and antioxidant scavenging [167,190,191].

As a result, immune cells migrating through inflamed tissues encounter continuously changing redox environments that differ markedly from those in non-inflamed regions [60,167,191,192].

Extracellular redox gradients modulate immune cell behavior in a distance-dependent manner. Immune cells in close proximity to inflammatory foci are exposed to higher ROS levels, resulting in increased oxidation of redox-sensitive surface proteins, altered receptor responsiveness, and changes in adhesion and migratory properties. By contrast, cells at the periphery of these gradients encounter lower, signaling-competent ROS concentrations that permit priming of activation without inducing overt oxidative damage. Collectively, these graded redox environments impose spatial control over immune cell activation and response dynamics [35,60,193,194,195].

Extracellular redox gradients occur alongside established inflammatory signaling systems, including chemokines, cytokines, and damage-associated molecular patterns (DAMPs) [196,197,198,199]. Oxidative modification of chemokines and cytokines alters their stability, receptor interactions, and diffusion properties, thereby influencing the spatial organization of inflammatory signals. In parallel, cytokine-driven activation states modulate local ROS production, linking inflammatory signaling to dynamic changes in extracellular redox conditions over time [35,60,200,201,202].

Extracellular redox gradients therefore do not act in isolation but are integrated into broader inflammatory signaling networks. The dominance of specific ROS-generating enzymes within a given tissue microenvironment determines whether redox gradients primarily support immune activation and coordination or drive pathological oxidative stress and tissue damage [35,167,192,202,203].

### 5.3. Redox Thresholds: Signaling Versus Damage

The effects of extracellular ROS depend on both concentration and exposure time. Extracellular redox activity, therefore, operates within thresholds that separate reversible regulatory modifications from irreversible oxidative damage [14,187].

At low to moderate levels, extracellular ROS primarily induce reversible oxidation of redox-sensitive residues in cell surface proteins and extracellular mediators. Such signaling-relevant redox conditions arise from spatially restricted and transient H_2_O_2_ production, for example, by DUOX1/2 at epithelial barriers or NOX1 at tissue interfaces. This localized oxidant generation permits reversible oxidation of redox-sensitive cysteine residues in membrane-associated receptors and adhesion molecules, thereby modulating receptor responsiveness, ligand binding, and cell–cell interactions without compromising tissue integrity. Within this concentration range, extracellular ROS contribute to the regulation of immune cell activation, directed migration, and intercellular communication [106,178,204].

When ROS levels exceed local buffering capacity or persist over time, extracellular redox reactions shift toward non-selective oxidation. This transition occurs when ROS production exceeds local antioxidant capacity, for example, due to sustained O_2_^•−^ generation by NOX2 in activated phagocytes or increased xanthine oxidase activity during hypoxia or ischemia–reperfusion. Excess oxidants induce oxidative modification of proteins, membrane lipids, and extracellular matrix components, including carbonylation, lipid peroxidation, and structural alteration of matrix proteins. These changes compromise tissue integrity and promote pro-inflammatory signaling through the release of damage-associated molecular patterns (DAMPs) [34,205,206,207]. Whether ROS act as signaling mediators or drivers of tissue injury is context-dependent and determined by the balance between enzymatic ROS sources and tissue-specific antioxidant capacity. Transient, spatially restricted ROS production supports immune defense, whereas persistent high-output ROS generation promotes sustained inflammation and progression toward chronic tissue damage [14,60,194,205].

## 6. Molecular Targets of Extracellular Redox Regulation in Immune Cells

Spatial confinement and threshold effects render extracellular redox regulation a selective, rather than diffuse, influence on immune responses. Redox conditions at the cell surface are interpreted through discrete molecular interfaces that link extracellular oxidative states to immune cell activation and inflammatory regulation.

### 6.1. Redox Regulation of Cell Surface Receptors

Cell surface receptors represent primary sites at which extracellular redox conditions are sensed and translated into intracellular signaling responses. Many immune receptors and associated signaling components contain redox-sensitive amino acid residues, particularly cysteines, whose oxidation state influences receptor conformation, ligand binding, and signal initiation.

**Figure 1:**
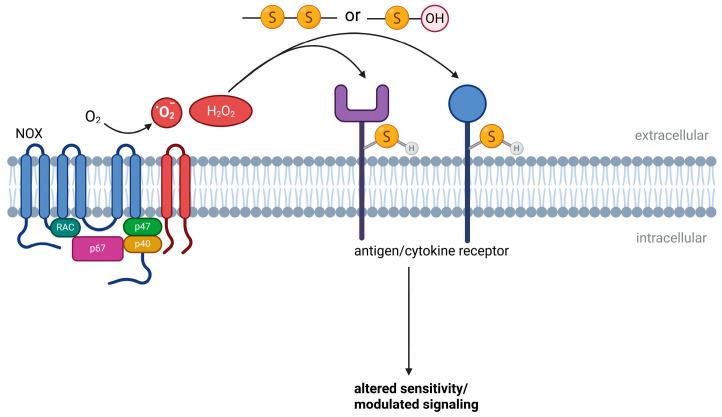
Redox regulation of cell surface receptors. NADPH oxidase (NOX)–derived extracellular ROS induce reversible oxidation of receptor-associated thiols, thereby tuning receptor sensitivity, signaling thresholds, and signal duration. Spatially restricted ROS production at the plasma membrane enables selective receptor modulation rather than binary on–off control. This figure was created using Biorender.com, Pollinger, K. (2026) https://app.biorender.com/citation/69709804233595ef801a7f35 (accessed on 19 February 2026). H_2_O_2_ = hydrogen peroxide; NOX = NADPH oxidase; O_2_ = molecular oxygen; O_2_^•−^ = superoxide; p67^phox^, p47^phox^, p40^phox^ = p67^phox^, p47^phox^, p40^phox^ proteins (subunits of NOX); RAC = RAC GTPase; -SH = thiol group; -S-OH = sulfene group; -S-S- = disulfide.

Extracellular ROS can modify receptor-associated thiols in a reversible manner, thereby tuning receptor sensitivity rather than inducing irreversible inactivation [167,208,209]. Such redox-dependent modulation has been described for antigen receptors, pattern recognition receptors, and cytokine receptors, where controlled oxidation alters signaling thresholds, amplification, or signal duration [204,208,209,210]. In this context, extracellular redox conditions act as modulators of receptor responsiveness rather than as binary on–off switches (Figure 1).

Redox regulation at the cell surface is facilitated by the close spatial coupling of ROS sources and receptors. NADPH oxidases and related oxidant-producing systems generate ROS in immediate proximity to the plasma membrane, allowing localized oxidation of receptor-associated residues while limiting diffusion into the bulk extracellular space. This spatial restriction favors selective receptor modulation over nonspecific oxidative damage [15,164,173,186,211]. In addition to direct receptor oxidation, extracellular ROS influence receptor function indirectly by modifying ligands or co-receptors. Oxidative changes in cytokines, chemokines, or adhesion molecules can alter receptor engagement and downstream signaling, further integrating extracellular redox conditions into immune activation pathways [60,106,194,212].

Taken together, redox-sensitive cell surface receptors constitute a critical interface through which extracellular oxidative states shape immune cell activation. By modulating receptor thresholds and signaling competence, extracellular ROS contribute to context-dependent immune responses without requiring widespread oxidative stress.

### 6.2. Redox Regulation of Cell Adhesion and Migration

Cell adhesion and migration are central to immune surveillance and inflammation and are highly sensitive to extracellular redox conditions. Adhesion molecules and cytoskeletal regulators exposed at the cell surface contain redox-sensitive residues whose oxidation state influences receptor affinity, clustering, and force transmission during cell movement.

Extracellular ROS modulate the activity of integrins and other adhesion receptors by altering thiol–disulfide balance within extracellular domains. Such redox-dependent modifications affect integrin activation states, ligand binding, and adhesion strength, thereby tuning immune cell arrest, spreading, and detachment. In this context, extracellular redox conditions influence not only whether immune cells adhere but also how dynamically they interact with endothelial surfaces and extracellular matrix components [213,214,215,216].

Redox regulation extends to migratory behavior within tissues. Local ROS gradients modify adhesion turnover and cytoskeletal coupling, shaping the balance between stable adhesion and directional migration. Low to moderate extracellular ROS levels support controlled migration by facilitating reversible adhesion remodeling, whereas excessive or sustained oxidative conditions impair motility by disrupting adhesion dynamics and promoting oxidative damage to membrane-associated proteins [214,216,217,218,219].

At the tissue level, redox-dependent effects on adhesion and migration influence how immune cells are distributed within inflamed environments. Changes in extracellular redox conditions affect leukocyte extravasation, interstitial movement, and retention at sites of inflammation, thereby shaping immune cell positioning. Through these effects on physical cell–cell and cell–matrix interactions, extracellular redox states modulate immune cell dynamics without directly determining effector functions [193,217].

### 6.3. Redox Regulation of Ion Channels and Transporters

**Figure 2:**
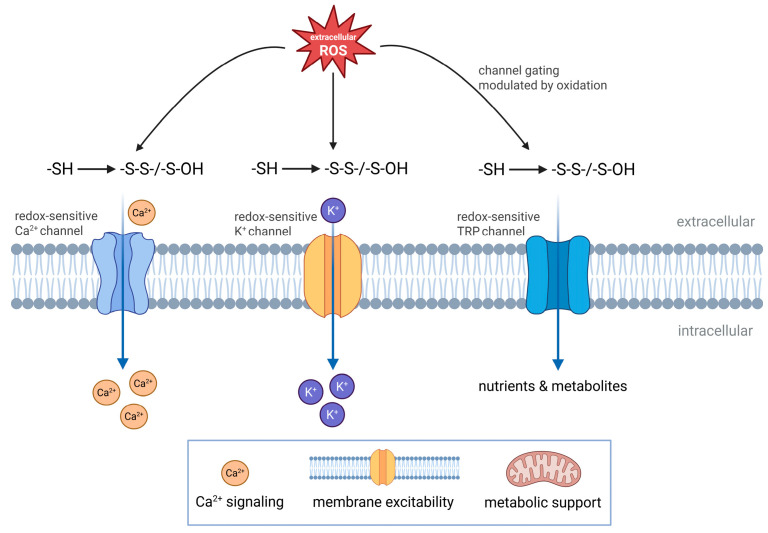
Redox regulation of ion channels and transporters. Extracellular ROS reversibly modify thiol residues on calcium, potassium, and transient receptor potential (TRP) channels or associated regulatory proteins, thereby modulating channel gating, conductance, and ionic homeostasis during immune cell activation. The Redox-dependent oxidation of membrane transporters further alters nutrient uptake, metabolite exchange, and pH regulation, linking extracellular redox conditions to cellular metabolism and signaling. Created with biorender.com, Pollinger, K. (2026) https://app.biorender.com/citation/69709804233595ef801a7f35 (accessed on 19 February 2026). Ca^2+^ = calcium ion; K^+^ = potassium ion; ROS = reactive oxygen species; -SH = thiol group; -S-OH = sulfene group; -S-S- = disulfide; TRP = transient receptor potential.

Ion channels and membrane transporters represent additional redox-sensitive targets through which extracellular oxidative conditions influence immune cell behavior. Many surface-exposed channels contain redox-reactive residues that couple changes in extracellular redox state to alterations in ion flux and membrane excitability (Figure 2).

Extracellular ROS modulate calcium, potassium, and transient receptor potential channels by reversible oxidation of channel-associated thiols or closely linked regulatory proteins. These modifications affect channel gating and conductance, thereby shaping calcium influx and ionic homeostasis during immune cell activation [220,221,222,223,224]. Redox sensitivity also extends to membrane transporters involved in nutrient uptake, metabolite exchange, and pH regulation. Oxidative modification of transporter proteins can alter transport efficiency and substrate availability, indirectly influencing metabolic and signaling capacity in inflammatory environments [14,178,187,209,225].

As for other cell surface targets, the effects of extracellular ROS on channels and transporters depend on concentration and exposure time. Transient redox changes support reversible functional modulation, whereas sustained oxidative conditions impair regulation of ion flux and contribute to cellular dysfunction [14,178,226].

## 7. Extracellular Redox Control of Immune Cell Activation

The molecular targets described above provide the interface through which extracellular redox conditions are integrated at the cellular level. These integrated redox inputs shape immune cell activation states and functional responses during inflammation.

### 7.1. Innate Immune Cells

Innate immune cells function within highly dynamic extracellular redox environments and are both sources and targets of ROS. During infection and tissue injury, neutrophils, monocytes, macrophages, and dendritic cells encounter rapidly changing redox conditions that influence activation, migration, and inflammatory output [53,60,194].

Extracellular ROS accompany pattern recognition receptor engagement, phagocytosis, and cytokine signaling, thereby modifying cell surface signaling and activation thresholds. The impact of these redox conditions differs among innate immune cell types. Neutrophils operate in and generate high extracellular ROS levels, whereas monocytes, macrophages, and dendritic cells respond primarily to lower, signaling-competent oxidative cues [53,56,60,106,227]. These differences in redox sensitivity contribute to functional specialization within the innate immune compartment and shape the early phases of inflammatory responses.

#### 7.1.1. Neutrophils

**Figure 3:**
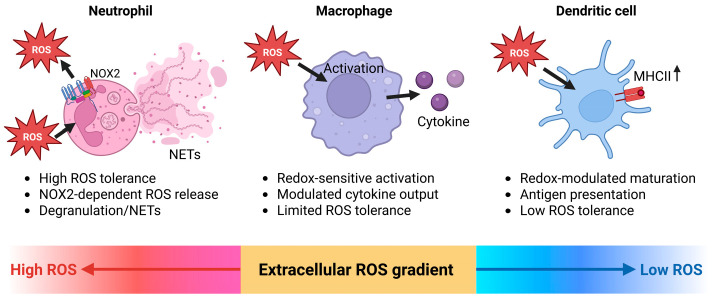
Extracellular redox control of innate immune cell activation. Neutrophils tolerate large amounts of ROS and also generate large amounts of extracellular ROS via NOX2 activation, thereby shaping the local redox environment. These high local ROS levels are associated with degranulation and neutrophil extracellular trap (NET) formation. Macrophages have limited ROS tolerance and locally generated ROS modulate activation state, cytokine production, and functional polarization. Dendritic cells encounter locally generated ROS that influence activation state, maturation, antigen presentation and cytokine output. They tolerate only low ROS concentrations. Created with biorender.com, Pollinger, K. (2026) https://app.biorender.com/citation/69709804233595ef801a7f35 (accessed on 19 February 2026). MHCII = major histocompatibility complex class 2; NETs = neutrophil extracellular trap; NOX2 = NADPH oxidase 2; ROS = reactive oxygen species.

Neutrophils are exposed to some of the highest extracellular ROS concentrations encountered by immune cells during acute inflammation. Upon recruitment to sites of infection or tissue damage, they generate large amounts of extracellular ROS through NOX2 activation [52,53,55,228], contributing to antimicrobial defense and shaping the local redox environment (Figure 3) [229,230,231].

Beyond pathogen killing, extracellular ROS influence neutrophil behavior in an autocrine and paracrine manner. Redox-dependent modification of cell surface proteins affects adhesion, spreading, and migratory dynamics, thereby regulating neutrophil accumulation and retention at inflammatory sites. These effects are closely linked to the spatial confinement of ROS production, allowing localized modulation without global loss of cell viability [54,60,232,233,234]. Extracellular redox conditions influence neutrophil effector behavior. High local ROS levels are associated with degranulation and neutrophil extracellular trap (NETs) formation [235,236,237,238], whereas lower or transient oxidative conditions modify activation thresholds and responses to chemotactic signals [229,239]. Compared with other innate immune cells, neutrophils remain functional under strong oxidative conditions, allowing their persistence at sites of acute inflammation [60,194]. The importance of robust NOX2-dependent ROS production for host defense is underscored by the severe infection susceptibility observed in individuals with NADPH oxidase deficiencies [56,240,241]. Sustained or excessive extracellular ROS exposure is associated with collateral tissue damage and prolonged inflammatory responses. ROS and myeloperoxidase-derived oxidants can modify host proteins, lipids, and nucleic acids, disrupt extracellular matrix integrity, and induce cellular dysfunction, thereby amplifying inflammation. In this context, neutrophils illustrate how extracellular redox activity can support antimicrobial defense while simultaneously contributing to tissue injury under inflammatory conditions [242,243,244].

#### 7.1.2. Monocytes and Macrophages

During recruitment and differentiation within inflamed tissues, monocytes and macrophages integrate extracellular redox cues that shape activation state, cytokine production, and functional polarization. In addition, these cells act as both sources and targets of extracellular ROS, placing them at the center of redox-regulated inflammatory circuits (Figure 3).

Extracellular ROS influence monocyte-to-macrophage differentiation and macrophage activation by adjusting signaling thresholds at the cell surface [60,245]. Low to moderate oxidative conditions are associated with changes in cytokine responsiveness and inflammatory output [194,246,247], whereas sustained oxidative stress impairs regulatory functions and promotes dysregulated activation [248,249]. Monocytes are particularly sensitive to elevated extracellular ROS levels, which can limit their survival and act as a regulatory mechanism controlling monocyte persistence within inflamed tissues. These effects are shaped by local antioxidant capacity and by the duration of ROS exposure [60,194,246].

Macrophage activation states are influenced by extracellular redox conditions. Oxidative tissue environments are commonly associated with increased inflammatory activity, whereas more balanced redox conditions accompany anti-inflammatory or tissue-reparative responses. Rather than defining discrete activation states, extracellular ROS are linked to gradual changes in macrophage function that mirror local tissue conditions [60,191,246,248]. Upon differentiation, macrophages acquire increased resistance to oxidative stress, enabling sustained function within ROS-rich environments while maintaining redox-sensitive control of cytokine production and polarization [250,251,252].

Monocytes and macrophages are less tolerant of high extracellular ROS levels than neutrophils. Prolonged oxidative exposure is associated with changes in cellular metabolism, reduced phagocytic capacity, and persistent inflammatory signaling. Redox-dependent modification of protein thiols contributes to these functional shifts, linking extracellular ROS exposure to longer-term changes in macrophage behavior. Under these conditions, extracellular redox states influence longer-term immune responses and tissue remodeling [195,246,248,253].

#### 7.1.3. Dendritic Cells

In dendritic cells, extracellular ROS encountered during tissue surveillance and migration influence maturation, antigen-presenting capacity, and cytokine output, thereby modulating their ability to initiate and shape adaptive immune responses (Figure 3).

Extracellular redox conditions influence dendritic cell function at the cell surface, including pattern recognition receptor signaling and costimulatory molecule expression. Changes in oxidative conditions are associated with altered antigen uptake and processing, while sustained oxidative stress is linked to impaired maturation and antigen presentation. The extent of these effects depends on local antioxidant capacity and the duration of ROS exposure [254,255,256,257]. Alterations in extracellular redox balance are associated with changes in cytokine production by dendritic cells and with differences in their capacity to prime T cells. Oxidative tissue environments correlate with distinct cytokine profiles and altered T cell responses [258,259]. In this context, dendritic cells respond to extracellular redox balance as part of their regulatory role rather than acting as direct effector cells [194].

### 7.2. Cells of the Adaptive Immunity

Adaptive immune cells are exposed to extracellular redox equilibrium during antigen-dependent activation and differentiation in inflamed tissues and secondary lymphoid organs. Under these conditions, redox balance influences activation thresholds and functional outcomes by modulating antigen receptor signaling, costimulatory interactions, and cytokine responsiveness.

#### 7.2.1. T Cells

T cells encounter extracellular redox equilibrium primarily during antigen-dependent activation at immune synapses and within inflamed tissues. In these contexts, local redox balance influences T cell activation thresholds by modulating T cell receptor signaling, costimulatory interactions, and early calcium-dependent signaling events.

Moderate extracellular oxidative conditions are associated with enhanced proximal T cell receptor signaling and altered sensitivity to costimulatory cues [3,260]. In contrast, sustained or excessive oxidative stress is linked to impaired T cell activation, reduced proliferative capacity, and altered cytokine production [261,262]. These effects reflect the sensitivity of T cell signaling components to changes in thiol redox state at the cell surface [3,260,261,263]. The extracellular redox equilibrium also affects T cell differentiation and effector function. Variations in redox balance correlate with differences in cytokine responsiveness and lineage-associated transcriptional programs, contributing to heterogeneity in effector T cell responses within inflamed environments [210,260,264,265].

T Cell Receptor (TCR) stimulation is coupled to oxidative signaling events that extend to the cell surface and the immune synapse. Controlled ROS generation accompanies TCR engagement and is required for effective proximal signaling, as antioxidant interference attenuates T cell activation [3,8,37,38,39,40]. Extracellular ROS modulate TCR-dependent signaling not by directly altering antigen recognition, but by influencing the thiol redox state of synapse-associated surface proteins and redox-sensitive phosphatases. This redox modulation occurs primarily through reversible oxidation of cysteine residues on key signaling proteins at the immunological synapse, including phosphatases such as the Src homology region 2 domain-containing phosphatase (SHP)-1, SHP-2, and CD45, which are critical for setting activation thresholds and controlling signal duration. Transient oxidation of these cysteine residues can inactivate phosphatase activity, thereby favoring kinase-driven signaling downstream of the TCR and adjusting both the threshold and persistence of T cell activation [210,266,267]. In inflamed tissues, extracellular redox equilibrium is therefore positioned to fine-tune TCR signaling strength and duration at the immune synapse [3,210,268].

##### Regulatory T Cells

**Figure 4:**
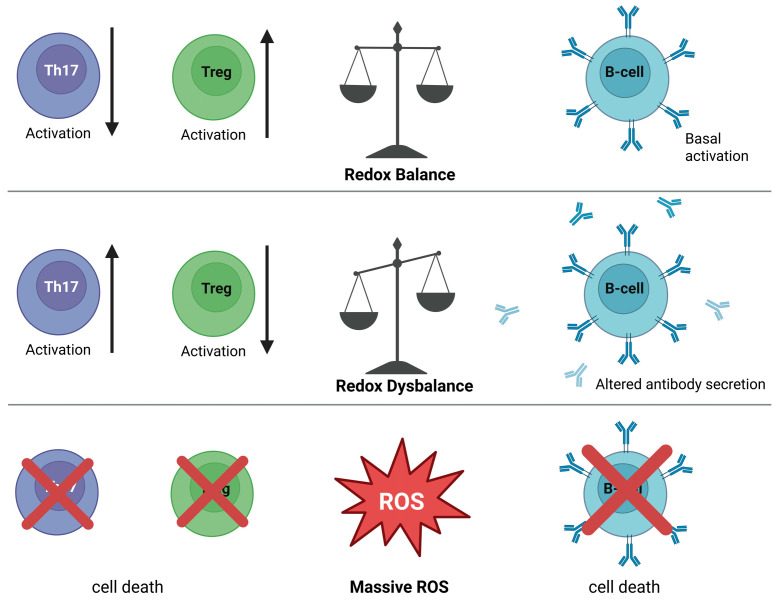
Cells of the adaptive immunity. The balance between Th17 cells and Tregs is sensitive to extracellular redox equilibrium. Under redox-stable conditions, activation of Th17 effector cells is low, and Tregs are highly activated. Whereas under oxidative conditions, Th17 cell activation is highly upregulated and Treg activation is downregulated. B cells show a basal activation, while redox balance and an altered antibody secretion under oxidative conditions. Massive ROS exposure leads to cell death of all adaptive immune cells. Created with biorender.com, Pollinger, K. (2026) https://app.biorender.com/citation/69709804233595ef801a7f35 (accessed on 19 February 2026). B cell = B lymphocyte; ROS = reactive oxygen species; Th17 = Th17 effector T cell; Treg = regulatory T cell.

Regulatory T cells (Treg) are susceptible to extracellular redox equilibrium. Their suppressive function is maintained in environments with tightly controlled redox balance and is altered when extracellular oxidative conditions become excessive or sustained. Physiological levels of extracellular ROS contribute to proper Treg differentiation and functional stability, whereas sustained redox imbalance disrupts regulatory activity. (Figure 4).

Changes in extracellular redox equilibrium are associated with modulation of Treg stability, signaling competence, and suppressive capacity. Oxidative stress can impair regulatory function by affecting cell surface signaling and cytokine responsiveness, whereas maintaining more balanced redox conditions supports the maintenance of the regulatory phenotype. Tregs exhibit an enhanced anti-oxidative capacity compared to conventional T cells, which supports their survival and function in moderate oxidative environments by preserving surface thiol balance and redox-sensitive signaling pathways. These effects are influenced by local antioxidant buffering and by competition with effector T cells within inflamed tissues [269,270,271,272]. Changes in the extracellular redox equilibrium are associated with shifts between regulatory and effector T cell activity. More oxidative environments tend to coincide with reduced regulatory function and increased effector T cell activity, whereas redox-stable conditions are more commonly observed in settings of immune regulation and resolution [166,269,270,273]. Beyond passive sensitivity, Tregs can actively influence the extracellular redox milieu by modulating thiol and cysteine availability in neighboring antigen-presenting and effector T cells, thereby reinforcing local immune regulation. Regulatory T cell function varies in relation to the extracellular redox equilibrium. Maintenance of suppressive activity is observed under redox-stable conditions, whereas sustained oxidative imbalance is associated with reduced regulatory control during immune responses [195].

##### Th17 Cells

Th17 cells are closely associated with inflammatory tissue environments characterized by altered extracellular redox equilibrium. Their differentiation and persistence are commonly observed under conditions in which oxidative cues accompany antigen stimulation and pro-inflammatory cytokine signaling (Figure 4).

Changes in the extracellular redox equilibrium correlate with Th17 polarization and functional stability. Oxidative environments are frequently associated with enhanced Th17 responses, whereas redox-stable conditions are less permissive for sustained Th17 activity. Rather than acting as direct instructive signals, extracellular redox states appear to shape the inflammatory context in which Th17 differentiation and effector function are maintained [194,274,275]. The balance between Th17 cells and regulatory T cells is particularly sensitive to extracellular redox equilibrium. Shifts toward oxidative tissue environments coincide with relative enrichment of Th17 responses, while redox-stable conditions favor regulatory dominance and attenuation of inflammation. In this way, extracellular redox imbalance contributes to the persistence of Th17-driven inflammation in chronic inflammatory settings [276,277,278,279].

#### 7.2.2. B Cells and Antibody Responses

B cells encounter the extracellular redox equilibrium during activation and differentiation in secondary lymphoid tissues and inflamed microenvironments. Under these conditions, the redox balance influences B cell receptor signaling thresholds, costimulatory interactions, and responsiveness to cytokines that support antibody production (Figure 4).

Changes in the extracellular redox equilibrium are associated with altered B cell activation and plasma cell differentiation. Oxidative extracellular environments correlate with modified B cell receptor signaling and altered antibody secretion, whereas redox-stable conditions are more compatible with sustained humoral responses. Importantly, these associations reflect properties of the surrounding extracellular redox milieu and are conceptually distinct from the localized, receptor-associated generation of ROS during B cell activation [280,281,282].

At the level of B cell activation, localized redox regulation at the cell surface further shapes signaling outcomes. Transient, NADPH oxidase-dependent ROS generation accompanies B cell receptor engagement and amplifies proximal signaling events that support activation and proliferation. In contrast, inhibition or genetic disruption of this localized ROS generation impairs B cell activation and reduces antibody responses [281,282].

During differentiation, B cells adapt to changing extracellular redox conditions by reinforcing antioxidant capacity, including glutathione- and thioredoxin-dependent buffering systems. This redox remodeling supports mitochondrial integrity, survival, and sustained antibody production, particularly in follicular B cell populations. Impaired antioxidant capacity, including defects in glutathione synthesis, is associated with compromised humoral immunity and increased susceptibility to infection [280].

The extracellular redox balance also shapes antibody responses indirectly by affecting antigen integrity and the availability of T cell help. Oxidative modification of extracellular antigens and proteins can alter immune recognition and influence the quality of humoral immunity [280,283,284].

## 8. Redox Crosstalk at Tissue Interfaces

While the extracellular redox equilibrium shapes immune cell activation at the cellular level, immune responses take place within complex tissue environments. At tissue interfaces, redox-dependent communication between immune cells, structural cells, and the extracellular matrix contributes to the spatial organization and outcome of inflammation.

### 8.1. Epithelial–Immune Redox Crosstalk

Epithelial barriers represent key sites of extracellular redox activity during immune responses. At mucosal and epithelial surfaces, immune cells encounter redox environments shaped by epithelial ROS production, local antioxidant capacity, and continuous exposure to microbial and environmental stimuli.

Epithelial cells generate extracellular ROS primarily through NADPH oxidases and DUOX enzymes localized at the apical membrane. These oxidant sources contribute to antimicrobial defense and barrier maintenance but also modify the extracellular redox equilibrium sensed by resident and infiltrating immune cells. Therefore, epithelial tissues act as active regulators of local immune responses rather than passive physical barriers. As a result, epithelial-derived ROS influence immune cell activation, migration, and retention at barrier surfaces [99,101,285,286,287,288]. Redox communication at epithelial–immune interfaces is bidirectional. Immune cells recruited to epithelial tissues generate ROS during activation that, beyond antimicrobial functions, can influence epithelial cell behavior and tissue repair processes. Conversely, epithelial-derived ROS modulate immune cell recruitment, activation, and polarization, thereby shaping local inflammatory responses [289].

Redox-dependent communication at epithelial–immune interfaces is spatially restricted and tightly regulated. Local antioxidant systems, extracellular thiols, and matrix components buffer ROS diffusion, limiting oxidative effects to the immediate vicinity of the epithelial surface. This confinement allows redox signals to modulate immune responses without inducing widespread tissue damage [60,175,290]. Disruption of epithelial redox control alters immune–epithelial interactions. Sustained oxidative imbalance at barrier surfaces is associated with impaired barrier integrity, enhanced immune cell infiltration, and persistent inflammation, as observed in chronic inflammatory diseases of epithelial tissues including inflammatory bowel disease and other barrier-associated pathologies [291,292,293].

### 8.2. Endothelial Redox Signaling and Immune Cell Recruitment

The vascular endothelium is a key interface at which extracellular redox processes regulate immune cell recruitment. Endothelial cells generate extracellular ROS in a spatially controlled manner, mainly through NOX enzymes at the plasma membrane, contributing to the regulation of vascular permeability and leukocyte trafficking during inflammation.

NOX-dependent extracellular ROS influence endothelial barrier function by modulating junctional organization and cytoskeletal dynamics. Transient oxidative signals are associated with reversible increases in vascular permeability, which facilitate immune cell extravasation [294,295,296], whereas sustained oxidative stress disrupts junction integrity and promotes prolonged inflammatory cell infiltration [297,298,299].

In addition to barrier regulation, endothelial redox signaling modulates leukocyte recruitment by influencing the expression and function of adhesion molecules and chemokine presentation. Endothelial-derived ROS regulate the activation and surface availability of key adhesion molecules, including vascular cell adhesion molecule-1 (VCAM-1) and intercellular adhesion molecule-1 (ICAM-1), which are essential for leukocyte tethering, firm adhesion, and transmigration during inflammation [300].

NOX-dependent extracellular ROS generation at the endothelial surface affects leukocyte adhesion and transmigration. Oxidative modification of endothelial adhesion molecules and changes in the redox state of the endothelial surface influence firm adhesion, diapedesis, and chemokine presentation, thereby shaping leukocyte recruitment [217,301]. Leukocyte–endothelial interactions can further enhance localized ROS production, promoting cytoskeletal rearrangement and transient junctional opening required for extravasation [302].

These processes are counterbalanced by endothelial antioxidant systems. Experimental inhibition of redox signaling reduces adhesion molecule-dependent leukocyte recruitment, highlighting the importance of endothelial redox control in inflammatory responses. Dysregulation of these mechanisms can lead to excessive leukocyte infiltration and tissue injury in chronic inflammatory conditions [300].

### 8.3. Extracellular Matrix as a Redox Scaffold

The extracellular matrix (ECM) provides a structural framework that also shapes extracellular redox conditions within tissues [303]. Matrix components influence the distribution, persistence, and local concentration of ROS by binding redox-active molecules, metals, and antioxidant enzymes. Due to its high content of long-lived structural proteins and comparatively limited antioxidant capacity, the ECM is particularly susceptible to oxidative modification during inflammatory conditions. Through these properties, the ECM contributes to spatially defined redox microenvironments at tissue interfaces.

Oxidative modification of ECM proteins alters their biochemical and mechanical properties, affecting cell adhesion, migration, and signaling. Changes in thiol–disulfide balance within matrix proteins, as well as oxidation of glycoproteins and proteoglycans, modify integrin engagement and growth factor availability. In addition, oxidative cleavage and cross-linking of ECM components can generate bioactive matrix fragments that further modulate immune cell recruitment and activation. These redox-dependent alterations influence how immune cells interact with the surrounding matrix during inflammation [174,303].

The ECM also acts as a reservoir for redox-active enzymes and antioxidants, including extracellular superoxide dismutase and metal-binding proteins [131,132]. Enzymes such as myeloperoxidase can associate with ECM components, locally concentrating oxidant production at sites of leukocyte infiltration. By buffering ROS diffusion and controlling redox gradients, matrix-associated factors limit the spatial spread of oxidative reactions and contribute to localized immune regulation [304].

During chronic inflammation or tissue injury, sustained oxidative imbalance leads to progressive ECM modification [305,306,307]. Such changes are associated with altered tissue architecture, impaired resolution of inflammation, and persistent immune cell activation [308,309]. Through cumulative redox-driven remodeling, the ECM can thus perpetuate inflammatory circuits and tissue dysfunction. Therefore, the ECM functions not only as a structural scaffold but also as a regulator of extracellular redox balance that shapes immune responses at the tissue level [174,306].

## 9. Extracellular Redox Imbalance in Inflammatory and Immune-Mediated Diseases

The tissue-level redox interactions described above acquire particular relevance when extracellular redox balance is persistently disturbed, a feature common to many inflammatory and immune-mediated diseases.

### 9.1. Acute Inflammation and Infection

Acute inflammatory responses are characterized by rapid and spatially confined alterations in extracellular redox balance. During infection or tissue injury, localized activation of NADPH oxidases in resident and infiltrating immune cells leads to transient increases in extracellular ROS that support pathogen clearance and early immune activation [53,60,230,310]. Professional phagocytes such as neutrophils and macrophages generate extracellular ROS via NOX2, producing O_2_^•−^ that is rapidly converted to H_2_O_2_ and further processed into highly microbicidal oxidants (e.g., hypochlorous acid), thereby directly contributing to antimicrobial defense [53].

Extracellular ROS support antimicrobial defense [7,310] and contribute to the coordination of immune cell recruitment and activation at sites of infection [242,311,312]. Redox-dependent modulation of endothelial permeability, chemokine activity, and immune cell adhesion facilitates leukocyte extravasation and tissue entry. By modifying proteins and signaling cues within the local extracellular environment, ROS further influence cytokine activity and endothelial barrier function. Concurrently, extracellular antioxidant systems restrict ROS diffusion, preserving spatial control and limiting collateral tissue damage [132,313].

When tightly regulated, these transient extracellular redox changes support effective host defense and resolution of inflammation [35,60,203,246]. However, excessive or prolonged extracellular ROS accumulation during acute infection can amplify tissue injury and delay resolution [242,306,314], illustrating the narrow window within which extracellular redox activity is beneficial in acute inflammatory settings [60,192,194,203]. The spatial confinement of extracellular ROS generation is therefore a key determinant of whether redox signaling supports pathogen clearance or promotes inflammatory tissue damage.

### 9.2. Chronic Inflammatory Diseases

Chronic inflammatory diseases are characterized by sustained disturbances of extracellular redox balance within affected tissues. In contrast to acute inflammation, extracellular ROS production in these conditions is prolonged, spatially expanded, and insufficiently counterbalanced by local antioxidant systems.

In diseases such as inflammatory bowel disease, rheumatoid arthritis, and asthma, persistent extracellular oxidative conditions alter immune–tissue interactions at barrier and stromal interfaces. Continuous NOX- and DUOX-dependent ROS generation modifies epithelial and endothelial function, extracellular matrix properties, and immune cell recruitment, thereby reinforcing inflammatory circuits rather than supporting resolution [103,315,316,317,318]. While intracellular ROS signaling and mitochondrial dysfunction critically contribute to inflammatory responses, chronic inflammation is additionally shaped by persistent alterations of extracellular redox conditions that directly affect tissue interfaces and intercellular communication [242].

Extracellular redox imbalance also affects immune cell composition and function in chronically inflamed tissues. Oxidative environments are associated with impaired regulatory mechanisms and sustained effector activity, contributing to ongoing cytokine production and tissue damage. Importantly, redox dysregulation in chronic inflammation does not represent a uniform oxidative stress response; rather, redox conditions can diverge between extracellular compartments and distinct immune cell subsets, with evidence that insufficient ROS availability in specific immune populations may likewise disrupt immune homeostasis and favor persistent inflammation. Rather than acting as a uniform oxidative stress response, these redox alterations remain locally structured and closely linked to disease-specific tissue contexts [60,194,195,306].

NOX2-dependent extracellular ROS production by infiltrating phagocytes represents a central mechanism driving sustained tissue damage and inflammatory amplification in chronic disease [319]. Persistent NOX2 activity promotes prolonged oxidation of extracellular matrix components, altered cytokine activity, and reinforcement of pro-inflammatory signaling pathways, thereby contributing to failure of inflammatory resolution [47,53]. These observations have positioned NOX2 as a therapeutic target in chronic inflammatory settings [320,321]. Pharmacological NOX inhibition and emerging isoform-selective strategies aim to attenuate pathological extracellular ROS production while preserving essential antimicrobial host defense, underscoring the translational relevance of spatially controlled NOX2 activity in chronic inflammation [52,320,322].

At the signaling level, persistent extracellular oxidative conditions converge on redox-sensitive inflammatory pathways, most prominently the NF-κB signaling axis. Extracellular ROS modulate NF-κB activity indirectly by shaping receptor-proximal signaling, cytokine availability, and redox-dependent regulation of kinases and phosphatases, thereby amplifying inflammatory gene expression in chronically inflamed tissues [8,37,38,323,324,325]. This positions NF-κB not as a direct redox target, but as an integrative signaling node through which extracellular redox imbalance is translated into sustained inflammatory transcriptional programs, providing an additional mechanistic link between redox dysregulation and chronic inflammation [323,326].

In parallel, chronic inflammatory diseases affecting epithelial barrier tissues are characterized by altered DUOX-dependent extracellular H_2_O_2_ production. DUOX1 and DUOX2 are prominently expressed at the apical surface of airway and gut epithelia, where they constitutively produce extracellular H_2_O_2_ in response to physiological stimuli [327]. Sustained or dysregulated DUOX activity at mucosal surfaces contributes to epithelial injury, impaired barrier integrity, and persistent immune activation by modifying extracellular redox conditions at the epithelial–immune interface. In gastrointestinal epithelia, DUOX2 expression is upregulated in patients with inflammatory bowel disease, consistent with altered mucosal redox homeostasis in chronic inflammation [328]. Animal models further demonstrate that loss of epithelial DUOX2 function alters host–microbiome interactions and attenuates colitis severity, implicating epithelial DUOX-dependent ROS in tissue injury and barrier dysfunction [102].

Over time, persistent extracellular oxidative conditions promote structural tissue changes, including barrier dysfunction [306,329], fibrosis, and altered cellular composition [316,330]. Through these effects, dysregulated extracellular redox balance contributes to the maintenance and progression of chronic inflammation across diverse disease entities [329,331].

### 9.3. Cancer and Tumor-Associated Immune Suppression

Tumor tissues are characterized by persistent alterations in extracellular redox balance that differ from both acute and resolving inflammatory environments. Sustained extracellular ROS production arises from tumor cells, stromal cells, and infiltrating immune cells and is only partially counterbalanced by local antioxidant systems.

Beyond effects on immune cells, extracellular redox imbalance in tumors also directly affects tumor cell–intrinsic signaling pathways that support malignant growth and persistence. Tumor cells, including cancer stem cell (CSC) populations, actively exploit redox regulatory mechanisms to maintain self-renewal capacity, therapy resistance, and metabolic adaptation within oxidative microenvironments. In this context, extracellular redox conditions interface with intracellular redox-sensitive transcriptional programs that govern stemness, metabolic reprogramming, and stress adaptation in CSCs. Altered extracellular redox conditions influence redox-sensitive signaling pathways in CSCs, modulate interactions with the tumor stroma, and contribute to the maintenance of stem-like phenotypes. Recent work highlights that redox regulation in CSCs integrates extracellular ROS availability with intracellular antioxidant responses and metabolic programs, thereby linking tumor redox balance to disease progression and therapeutic resistance [332].

Within the tumor microenvironment, extracellular redox imbalance contributes to immune suppression by affecting multiple immune cell populations. Oxidative conditions are associated with impaired effector T cell activation, reduced antigen-presenting capacity of dendritic cells, and relative enrichment of regulatory immune programs. These effects reflect altered redox conditions at cell surfaces and tissue interfaces rather than uniform intracellular oxidative stress [333,334,335]. Therefore, extracellular ROS act as permissive factors that support the expansion and functional dominance of immunosuppressive cell populations, including regulatory T cells and myeloid-derived suppressor cells, which are known to produce and respond to oxidative signals within tumors [336].

The extracellular redox imbalance also influences the spatial organization of immune cells within tumors. High levels of ROS in the tumor microenvironment are associated with immunosuppression and impaired effector T cell function, contributing to altered immune infiltration and dysfunctional immune cell accumulation. Aberrant stromal and chemokine profiles in the tumor milieu further restrict lymphocyte penetration and promote immune exclusion [337]. ROS-dependent modification of endothelial function, extracellular matrix components, and chemokine activity contributes to these spatial constraints, reinforcing immune cell exclusion or retention of dysfunctional immune subsets within tumor tissue [338,339].

**Figure 5:**
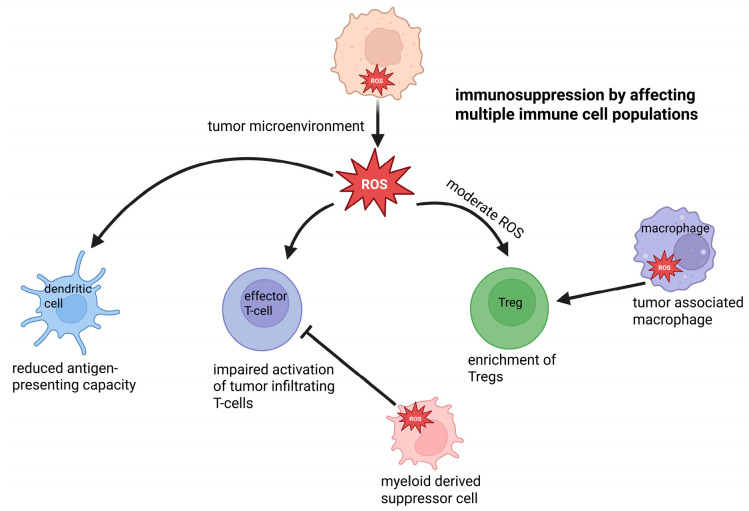
Cancer and Tumor-Associated Immune Suppression. Extracellular redox imbalance in the tumor microenvironment contributes to immunosuppression by affecting multiple immune cell populations. Oxidative conditions lead to reduced antigen-presenting capacity of dendritic cells, impaired effector T cell activation, and to relative enrichment of Tregs under moderate ROS conditions. Myeloid-derived suppressor cells also contribute to elevated ROS in tumors and inhibit effector T cell activity. ROS generated by tumor-associated macrophages additionally lead to enrichment of Tregs. Created with biorender.com, Pollinger, K. (2026) https://app.biorender.com/citation/69709804233595ef801a7f35 (accessed on 19 February 2026). ROS = reactive oxygen species; Treg = regulatory T cell.

Thus, elevated ROS in the tumor microenvironment are associated with tumor-induced immunosuppression and impaired immune surveillance, reflecting a disrupted redox balance that promotes dysfunction of tumor-infiltrating lymphocytes and favors regulatory and suppressive immune cell phenotypes, thereby contributing to an immune-evasive microenvironment [337]. In addition, infiltrating myeloid-derived suppressor cells and regulatory T cells contribute to elevated ROS in tumors, which can inhibit effector T cell activity and sustain an immune-evasive environment (Figure 5) [340].

### 9.4. Sepsis and Systemic Redox Dysregulation

**Figure 6:**
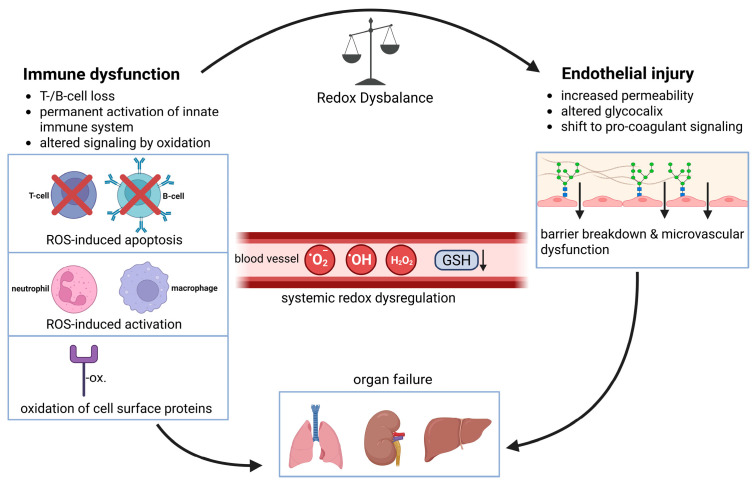
Sepsis and systemic redox dysregulation. In sepsis, excessive extracellular ROS generated by activated immune cells exceed systemic antioxidant capacity, resulting in widespread redox imbalance. Oxidative stress contributes to immune dysfunction by promoting lymphocyte apoptosis, inducing prolonged activation of innate immune cells, and interfering with redox-sensitive signaling pathways by oxidation of cell surface proteins. In parallel, endothelial cells undergo oxidative injury, leading to barrier disruption, microvascular dysregulation, and a shift toward pro-coagulant signaling. Together, immune and endothelial dysfunction form a self-reinforcing process that impairs tissue perfusion and promotes organ injury and progression to multi-organ failure. Created with biorender.com, Pollinger, K. (2026) https://app.biorender.com/citation/69709804233595ef801a7f35 (accessed on 19 February 2026). GSH = reduced glutathione; H_2_O_2_ = hydrogen peroxide; O_2_^•−^ = superoxide; OH^•^ = hydroxyl radical; ox. = oxidation; ROS = reactive oxygen species.

Sepsis represents an extreme manifestation of inflammatory redox imbalance, in which locally controlled extracellular redox signaling collapses into a systemic disturbance. In contrast to the spatially confined redox alterations observed in tumors or chronic inflammatory tissues, sepsis is characterized by widespread extracellular ROS generation in the circulation and at vascular interfaces, exceeding the capacity of antioxidant buffering systems and disrupting immune, endothelial, and metabolic homeostasis (Figure 6) [341,342,343].

During sepsis, excessive extracellular ROS originate predominantly from activated neutrophils and macrophages, as well as from dysfunctional mitochondria in immune, endothelial, and parenchymal cells. Mitochondrial dysfunction is driven by inflammatory mediators, hypoxia, nitric oxide, and peroxynitrite stress, and impaired electron transport chain activity, resulting in increased electron leakage and ROS formation. These mitochondrial ROS sources are only insufficiently counterbalanced by systemic antioxidant defenses. As a result, redox homeostasis is lost across the vascular compartment and multiple organ systems (Figure 6) [344,345,346].

This systemic redox dysregulation affects the function of immune cells, vascular integrity, and organ perfusion [347,348,349]. Rather than supporting coordinated immune defense, excessive extracellular oxidative stress contributes to immune paralysis, endothelial dysfunction, and tissue injury [347,348], thereby linking redox imbalance to both the hyper-inflammatory and immunosuppressive phases of the septic response (Figure 6) [348,350].

At the level of immune cell regulation, systemic oxidative stress in sepsis is associated with profound alterations in cell survival, metabolism, and cytokine signaling [351]. Excessive redox stress promotes apoptosis of lymphocyte populations, including T and B cells, thereby contributing to impaired adaptive immune responses and reduced pathogen clearance. In parallel, apoptosis of neutrophils and monocytes is delayed, resulting in prolonged survival of activated innate immune cells that sustain inflammatory tissue injury [13,348,351,352,353]. Redox-dependent post-translational modifications of signaling proteins further disrupt innate and adaptive immune signaling by altering kinase–phosphatase balance and transcriptional control. Together, these mechanisms facilitate the transition from an early hyper-inflammatory phase to a subsequent state of immunosuppression characteristic of sepsis (Figure 6) [346].

Endothelial injury is a key consequence of systemic redox imbalance in sepsis. During sepsis, endothelial cells are both a target and source of inflammation and oxidative stress, with excessive ROS and nitrogen species contributing to endothelial activation and dysfunction. Endothelial cells produce ROS and nitrogen species, contribute to microvascular permeability changes, and are central to dysregulated immune-endothelial interactions [354]. Extracellular ROS contribute to endothelial dysfunction by damaging the endothelial surface layer and facilitating the formation of reactive nitrogen species such as peroxynitrite, which can be generated when nitric oxide reacts with O_2_•^−^ during sepsis [355]. These redox-dependent alterations are associated with increased vascular permeability and microvascular dysregulation, including disruption of the endothelial glycocalyx and impaired barrier integrity, which correlate with altered blood flow and interstitial leakage [355]. Moreover, systemic endothelial activation in sepsis is linked to a shift toward a pro-coagulant phenotype, with endothelial expression of adhesion molecules and pro-thrombotic signaling contributing to microvascular thrombosis and impaired circulation [354]. Importantly, endothelial dysfunction emerges early during sepsis and correlates with impaired tissue perfusion, organ dysfunction, and progression toward multi-organ failure (Figure 6) [356].

Together, these observations support the view that systemic redox dysregulation in sepsis is clinically relevant, since oxidative stress co-occurs with immune dysfunction and endothelial injury and is associated with disease severity and outcome. Biomarkers of oxidative stress and impaired antioxidant capacity have been reported to correlate with severity and mortality in sepsis [357,358,359]. Rather than reflecting isolated oxidative injury, excess ROS contribute to a coupled failure mode in which immune suppression (including lymphocyte loss and dysfunction) and endothelial barrier/microvascular dysfunction reinforce each other, promoting tissue hypoperfusion and organ injury [341,342,347,348].

## 10. Conclusions

The extracellular redox balance represents an important, yet still incompletely understood, determinant of immune cell activation and inflammatory regulation. Beyond their established roles as intracellular signaling mediators or sources of oxidative damage, ROS in the extracellular space act within spatially and temporally constrained environments that influence immune responses at tissue interfaces. These redox conditions shape immune cell behavior primarily by modulating surface-associated signaling events, receptor sensitivity, and intercellular communication rather than by inducing nonspecific oxidative stress.

As outlined in this review, extracellular redox regulation operates as a selective and context-dependent mechanism. Localized ROS production, redox gradients, and extracellular antioxidant buffering define thresholds for immune activation, migration, and effector function. Under physiological conditions, these processes support host defense, tissue repair, and coordinated immune responses. When extracellular redox control is disturbed, however, oxidative environments shift from regulatory to pathogenic, contributing to sustained inflammation, immune dysfunction, and tissue injury.

From a translational perspective, these insights highlight why broadly acting antioxidant interventions have shown limited benefit in inflammatory diseases. Rather than overall oxidative load, loss of spatial and temporal redox control emerges as a critical determinant of pathology. Consequently, therapeutic strategies that aim to restore localized extracellular redox regulation—such as isoform-specific modulation of ROS-producing enzymes, reinforcement of barrier-associated redox buffering, or indirect modulation of redox-sensitive inflammatory signaling pathways—may offer greater specificity and efficacy than global suppression of reactive species.

Progress in this area is limited by technical challenges. Direct assessment of extracellular redox processes in vivo with sufficient spatial and temporal resolution remains difficult, and many current approaches rely on indirect or bulk measurements that poorly resolve localized redox gradients at cell surfaces. In addition, available detection systems often lack the sensitivity and compartmental specificity required to distinguish extracellular from intracellular ROS signals in complex tissue environments. Furthermore, tools enabling selective modulation of extracellular redox states without affecting intracellular signaling are still limited, restricting mechanistic dissection of compartment-specific redox functions.

Future studies should therefore focus on defining redox-sensitive targets at the cell surface, mapping extracellular redox conditions in vivo, and assessing how redox modulation with spatial and temporal specificity influences immune function. Such approaches may enable the development of interventions that preserve essential redox-dependent host defense mechanisms while limiting chronic inflammatory tissue damage, while complementing established intracellular and tissue-specific redox regulatory models by extending redox control to the extracellular space and tissue interfaces.

In summary, extracellular redox regulation constitutes a distinct layer of immune control with direct relevance for inflammatory pathology and therapeutic intervention.

## Figures and Tables

**Table 1:** Determinants and functional consequences of extracellular redox gradients during inflammation.

Feature	Determinant	Characteristics of the Gradient	Functional Consequences for Immune Cells
**Source localization **	NADPH oxidases (NOX2), DUOX1/2, xanthine oxidase	Focal and steep ROS release at sites of tissue injury or infection	Localized immune cell activation, enhanced adhesion and retention at inflammatory foci
**Diffusion constraints**	Short ROS half-life, interaction with extracellular matrix components	Rapid decay over micrometer-scale distances	Distance-dependent priming and activation of immune cells
**Antioxidant buffering**	Extracellular SOD3, catalase, plasma thiols	Gradient steepness shaped by local scavenging capacity	Limitation of oxidative damage at the periphery of inflamed tissues
**Chemical identity of ROS**	O_2_^•−^, H_2_O_2_, secondary oxidants	Signaling-competent versus damage-inducing gradients	Reversible redox signaling versus oxidative tissue injury
**Temporal dynamics**	Transient versus sustained ROS production	Pulsed or persistent redox gradients	Acute immune activation versus chronic inflammatory responses
**Integration with inflammatory mediator**	Redox modification of chemokines and cytokines	Altered stability, receptor interaction, and diffusion	Reshaping of immune cell recruitment and spatial organization

## Data Availability

No new data were created or analyzed in this study. Data sharing is not applicable to this article.
